# Biosensor-Coupled *In Vivo* Mutagenesis and Omics Analysis Reveals Reduced Lysine and Arginine Synthesis To Improve Malonyl-Coenzyme A Flux in *Saccharomyces cerevisiae*

**DOI:** 10.1128/msystems.01366-21

**Published:** 2022-03-01

**Authors:** Chenxi Qiu, Mingtao Huang, Yishan Hou, Huilin Tao, Jianzhi Zhao, Yu Shen, Xiaoming Bao, Qingsheng Qi, Jin Hou

**Affiliations:** a State Key Laboratory of Microbial Technology, Shandong University, Qingdao, People’s Republic of China; b School of Food Science and Engineering, South China University of Technology, Guangzhou, People’s Republic of China; c State Key Laboratory of Biobased Material and Green Papermaking, School of Bioengineering, Qi Lu University of Technology, Jinan, People’s Republic of China; UiT–The Arctic University of Norway

**Keywords:** malonyl-CoA biosensor, growth-based screening, *in vivo* mutagenesis, omics analysis, *Saccharomyces cerevisiae*

## Abstract

Malonyl-coenzyme A (malonyl-CoA) is an important precursor for producing various chemicals, but its low availability limits the synthesis of downstream products in Saccharomyces cerevisiae. Owing to the complexity of metabolism, evolutionary engineering is required for developing strains with improved malonyl-CoA synthesis. Here, using the biosensor we constructed previously, a growth-based screening system that links the availability of malonyl-CoA with cell growth is developed. Coupling this system with *in vivo* continuous mutagenesis enabled rapid generation of genome-scale mutation library and screening strains with improved malonyl-CoA availability. The mutant strains are analyzed by whole-genome sequencing and transcriptome analysis. The omics analysis revealed that the carbon flux rearrangement to storage carbohydrate and amino acids synthesis affected malonyl-CoA metabolism. Through reverse engineering, new processes especially reduced lysine and arginine synthesis were found to improve malonyl-CoA synthesis. Our study provides a valuable complementary tool to other high-throughput screening method for mutant strains with improved metabolite synthesis and improves our understanding of the metabolic regulation of malonyl-CoA synthesis.

**IMPORTANCE** Malonyl-CoA is a key precursor for the production a variety of value-added chemicals. Although rational engineering has been performed to improve the synthesis of malonyl-CoA in S. cerevisiae, due to the complexity of the metabolism there is a need for evolving strains and analyzing new mechanism to improve malonyl-CoA flux. Here, we developed a growth-based screening system that linked the availability of malonyl-CoA with cell growth and manipulated DNA replication for rapid *in vivo* mutagenesis. The combination of growth-based screening with *in vivo* mutagenesis enabled quick evolution of strains with improved malonyl-CoA availability. The whole-genome sequencing, transcriptome analysis of the mutated strains, together with reverse engineering, demonstrated weakening carbon flux to lysine and arginine synthesis and storage carbohydrate can contribute to malonyl-CoA synthesis. Our work provides a guideline in simultaneous strain screening and continuous evolution for improved metabolic intermediates and identified new targets for improving malonyl-CoA downstream product synthesis.

## INTRODUCTION

Saccharomyces cerevisiae is a promising microbial cell factory that has been used to produce diverse value-added chemicals (1, 2). Malonyl-coenzyme A (malonyl-CoA) is an important intracellular metabolite in the production of a variety of chemicals such as polyketides, flavonoids, and biofuels (3–7). However, its intracellular availability is often limited in S. cerevisiae because of the tight regulation of the pathway and the competition with cellular metabolism. Therefore, it is highly desirable to enhance the malonyl-CoA availability to improve the synthesis of its downstream products. Although rational engineering approaches have been performed to increase the malonyl-CoA level (8), the cellular metabolism machinery is complex, and thus it is still required to rapidly develop effective approaches for generating mutant strains with much more improved malonyl-CoA availability and identify new targets for further engineering.

Biosensors is a powerful tool to link the metabolite concentration with selectable phenotype for high-throughput screening (9, 10). The screening of the phenotype such as fluorescence depends on expensive equipment like fluorescence-activated cell sorting (FACS) or droplet-based microfluidics (11, 12). Alternatively, coupling the metabolite concentration to growth-selectable phenotype offers a valuable complementary tool for high-throughput screening. Growth-based screening can enrich the beneficial mutations easily, which will largely facilitate the selection. Despite this, the number of successful growth-based designs is very limited in yeast. These limited growth-based designs are mainly used to find key enzymes in specific pathway (13), and very few studies have focused on screening the genome-wide mutagenesis library. Therefore, growth-based screening, coupled with rapid genome-wide mutagenesis, can circumvent the labor-intensive screening or the requirement for expensive equipment and obtain the desired phenotypes very quickly.

Compared to traditional mutagenesis approaches such as physical and chemical mutagenesis and the construction of the genome modification library (14) or gene regulation library (15, 16), *in vivo* mutagenesis can achieve continuous mutagenesis during the cultivation (17). Coupling *in vivo* mutagenesis with adaptive evolution linked continuous evolution and selection simultaneously and thus has significantly accelerated evolution. When combined with omics analysis, a key mechanism can be identified to contribute to specific phenotypes. *In vivo* mutagenesis has been employed to improve stress tolerance and the production of metabolites such as *n*-butanol, lysine, tyrosine, and isoprenoid in Escherichia coli (17–20). However, the application of error-prone replication for cell factory construction is relatively rare in Saccharomyces cerevisiae.

**FIG 1:**
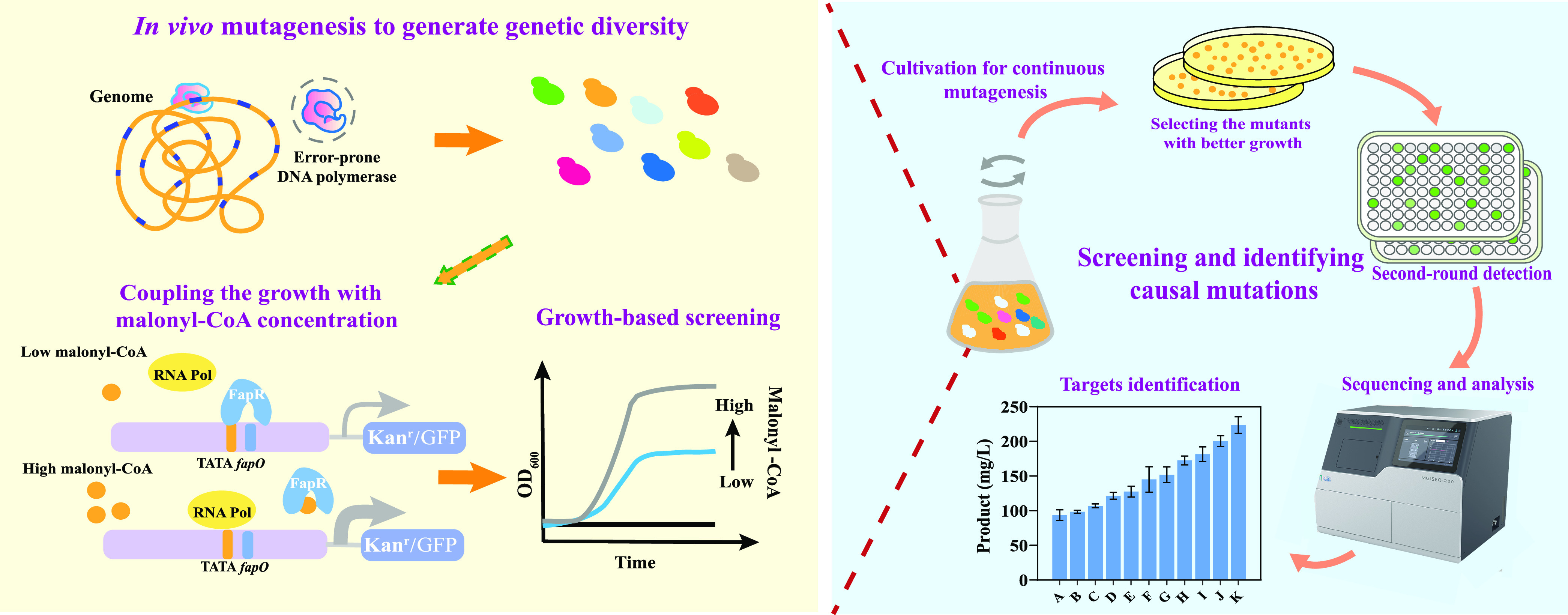
Construction scheme of the growth-based selection system and *in vivo* evolution to improve malonyl-CoA flux in S. cerevisiae. An error-prone DNA polymerase was used to construct the *in vivo* evolution system. Two reporter genes (*KanMX* and *GFP*) were expressed under the control of malonyl-CoA sensing circuits to develop the growth-based selection and subsequent screening system. The mutagenesis library was generated by cell subculture, and the culture was then spread on plates containing 800 mg/L G418 for growth-based selection. The colonies with better growth were selected, and their fluorescence was determined to obtain mutant strains with high malonyl-CoA availability. Subsequently, whole-genome sequencing, transcriptome analysis, and reverse engineering were performed to identify beneficial gene targets.

Here, using malonyl-CoA biosensors we have previously constructed (21, 22), we created a growth-based screening system that linked the metabolite concentration to cell growth in S. cerevisiae. Subsequently, we coupled it with replication-assisted evolution to develop strains with improved malonyl-CoA availability (Fig. 1). Using this growth-based screening system, we selected mutant strains with improved malonyl-CoA flux and identified the key targets related to malonyl-CoA availability by whole-genome sequencing, transcriptome analysis, and reverse engineering. The new process especially altered carbohydrate storage, and weakening lysine and arginine synthesis was found to improve malonyl-CoA flux. Thus, this study not only developed a useful approach for generating increased metabolite production but also improved our understanding of the metabolic pathway regulation of malonyl-CoA and enabled strain improvement. Since malonyl-CoA is a representative central metabolite, we believe the approach developed here should be readily applicable for developing strains that produce other metabolites.

## RESULTS

### Design of growth-based screening system.

In our previous study, using the FapR/*fapO* system, we developed two malonyl-CoA biosensors to enable transcription repression or activation in response to changes in the malonyl-CoA level (21, 22). To construct a growth-coupled circuit, we introduced either a toxicity gene or an antibiotic resistance gene to the biosensor to replace the *GFP* gene as an actuator in this study. In the first circuit, the cytosine deaminase encoding gene, *FCY1*, was expressed under the control of a repressive biosensor (FapR-Med2/LEU2p·1*fapO-FCY1) (22). Cytosine deaminase converts 5-fluorocytosine to 5-fluorouracil, which is toxic to the cell because it interferes with RNA synthesis (13, 23). We have previously demonstrated that in the presence of FapR-Med2, a high malonyl-CoA level repressed the transcription of the LEU2p·1*fapO promoter (22). Therefore, a high concentration of malonyl-CoA should reduce the expression of cytosine deaminase and increase the growth rate of the strain (see Fig. S1a in the supplemental material). However, the addition of 5-fluorocytosine has no effect on the growth of *FCY1*-deficient strain (see Fig. S1b). Different concentrations of 5-fluorocytosine (1, 3, and 5 mg/L) were added to the medium, and the effect on the growth rate was determined (see Fig. S1c to e). As expected, the growth rate of the strain expressing FapR-Med2 was lower than that of the strain without FapR-Med2, especially with 3 mg/L 5-fluorocytosine. However, the growth rate difference between these strains was not significant enough; hence, this circuit does not meet the requirement for growth-based selection.

10.1128/msystems.01366-21.1 FIG S1 Design of a growth-based screening system using malonyl-CoA repressive sensor in yeast. (a) The expression of *FCY1* was controlled by LEU2p·1*fapO promoter. When intracellular malonyl-CoA level is low, the FapR-Med2 binds to the promoter and activates the transcription of *FCY1*; when intracellular malonyl-CoA level is high, the FapR-Med2 dissociates from the promoter and reduces transcription of *FCY1*. Fcy1 converts 5-fluorocytosine to 5-fluorouracil, which is toxic to the cells; therefore, low expression of *FCY1* mitigates the toxicity to fluorouracil. (b) Growth curves of Δ*FCY1* strains in the presence of concentrations of 1, 3, and 5 mg/L 5-fluorocytosine. (c to e) Growth curves of strains with or without FapR-Med2 in the presence of concentrations of 1, 3, and 5 mg/L 5-fluorocytosine, respectively. The control is the background strain empty plasmid. Error bars represent the means ± the standard deviations from three independent experiments. Download FIG S1, EPS file, 1.5 MB. Copyright © 2022 Qiu et al. 2022 Qiu et al. https://creativecommons.org/licenses/by/4.0/ This content is distributed under the terms of the Creative Commons Attribution 4.0 International license.

**FIG 2:**
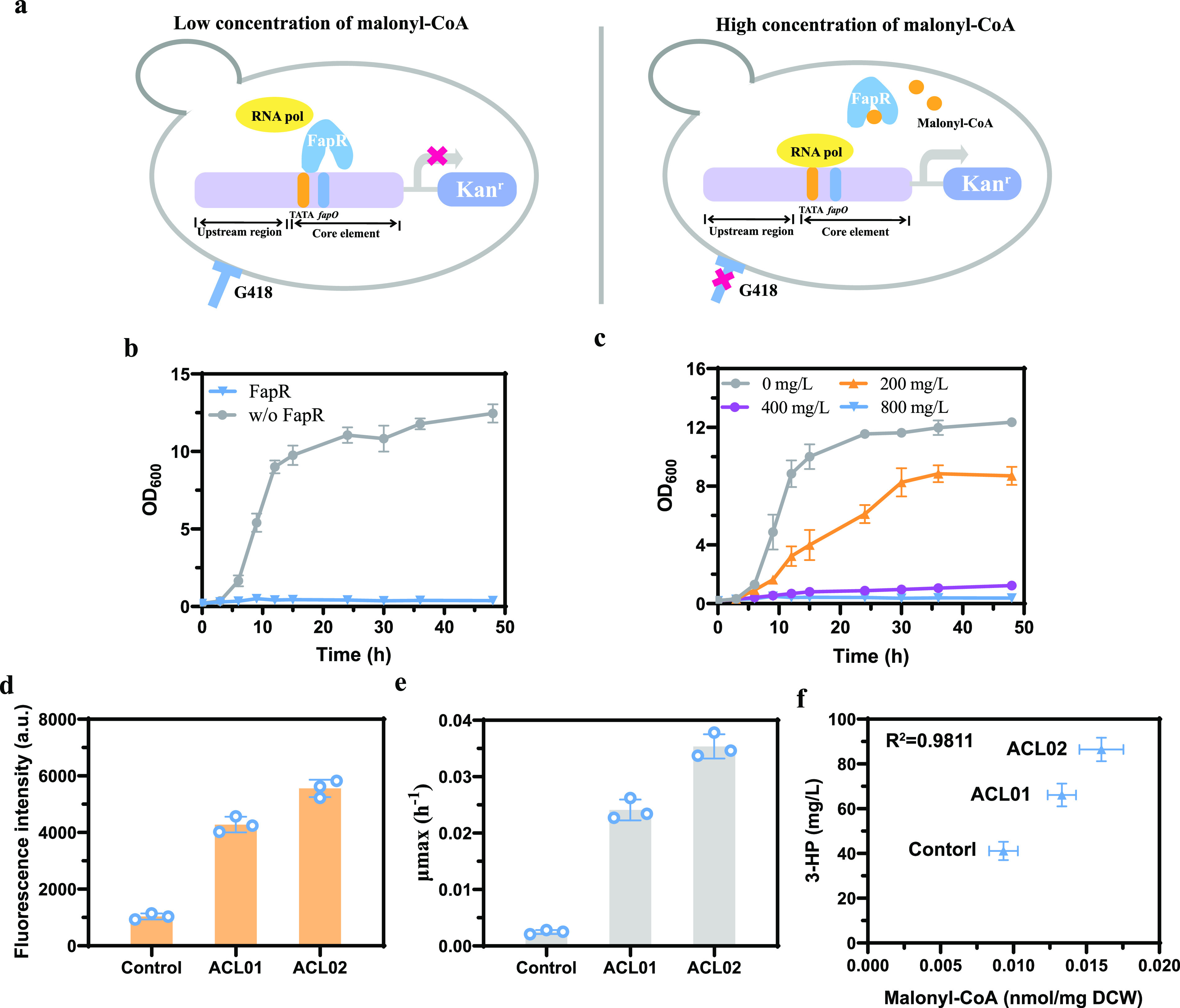
Design of a growth-based circuit using a malonyl-CoA activated sensor in yeast. (a) Principle of a growth-based circuit that senses the malonyl-CoA concentration. When the intracellular malonyl-CoA level is low, FapR binds to the promoter and blocks the expression of *KanMX*, and the strain cannot survive on G418 containing plates; when the intracellular malonyl-CoA level is high, FapR dissociates from the promoter and the expression of KanMX is activated, giving the strain G418 resistance. (b) Growth of the strain with or without FapR in the presence G418 resistance. (c) The circuit containing strain showed growth differences in the presence of different concentrations of G418. (d) Fluorescence intensities of different malonyl-CoA producers—ACL01 (CEN.PK2-1C, *icl1*::*loxP*, Detal15::*loxp-TEF1p-YHM2-ADH1t*, pYlACL) and ACL02 (CEN.PK2-1C, *icl1*::*loxP*, Detal15::*loxp-TEF1p-CTP1-ADH1t/PGK1p-OAC1-CYC1t*, pYlACL)—and the control strain expressing the pJfpaO-GFP-fapR plasmid. Fluorescence intensity was normalized to the OD measured at 600 nm. (e) Maximum specific growth rates of ACL01, ACL02, and control strain expressing the pJfapO-fapR-Kan^r^ plasmid in the presence of 800 mg/L G418. (f) Correlation of intracellular malonyl-CoA concentration and 3-HP titer of ACL01, ACL02, and control strains expressing the pJFE3-Mcr plasmid. The control was CEN.PK2-1C. Error bars represent the means ± the standard deviations of three independent experiments.

In another design, we used the malonyl-CoA activation circuit to regulate the expression of the *kanMX* gene, a widely used antibiotic resistance gene in S. cerevisiae (FapR/*TEF1up-fapOGAL1pcore-kanMX*) (24) (Fig. 2a). Previously, we have shown that FapR bound to the malonyl-CoA-responsive hybrid promoter, *TEF1up-fapOGAL1pcore*, to inhibit transcription, but when the malonyl-CoA level increased, FapR dissociated from the promoter to activate the transcription. Therefore, the transcription level of *KanMX* should correlate with the malonyl-CoA level and the growth rate under G418 exposure (Fig. 2a). There was a notable difference in the growth rate between the strains with or without FapR in the presence of G418 (Fig. 2b). After different concentrations of G418 were added to the medium, the growth rate of the strain containing FapR/*TEF1up-fapOGAL1pcore-kanMX* was significantly inhibited; it was completely inhibited by 800 mg/L G418 (Fig. 2c).

To test the efficiency of this circuit, we evaluated the growth of the strains with different levels of malonyl-CoA in the medium with 800 mg/L G418. Although cerulenin addition has been reported to increase the malonyl-CoA level, it also affected the growth and thus cannot be used for growth-based tests (25). Therefore, two strains, ACL01 and ACL02, with different malonyl-CoA levels were constructed by expressing citrate lyase (ACL) and engineering citrate transports (see Fig. S2a to c) (26–28). We compared the fluorescence intensity, the maximum specific growth rate in the presence of G418, and the production of 3-hydroxypropionic acid (3-HP), a malonyl-CoA-derived chemical (Fig. 2d to f). The maximum specific growth rate of the two strains indeed correlated with the fluorescence and 3-HP production (Fig. 2d to f). We also observed a positive correlation between 3-HP production and intracellular malonyl-CoA concentration (Fig. 2f), which demonstrates that the growth rate was coupled to the intracellular malonyl-CoA level. Therefore, this circuit can link the metabolite concentration with the growth phenotype and therefore meets the requirements for screening.

10.1128/msystems.01366-21.2 FIG S2 Growth of strains with different malonyl-CoA fluxes. (a) Schematic view of metabolic pathway of two strains with different malonyl-CoA levels. (b) Growth curves of ACL01, ACL02, and control strains in without 800 mg/L G418. (c) Intracellular concentration of malonyl-CoA of ACL01, ACL02, and control strains. Error bars represent the means ± the standard deviations from three independent experiments. Download FIG S2, EPS file, 1.3 MB. Copyright © 2022 Qiu et al. 2022 Qiu et al. https://creativecommons.org/licenses/by/4.0/ This content is distributed under the terms of the Creative Commons Attribution 4.0 International license.

### Establishment of an *in vivo* mutagenesis system.

To achieve *in vivo* evolution, we expressed the error-prone DNA polymerase δ (polδ) in S. cerevisiae to increase the mutation rate (29). Previous studies have demonstrated that the mutations D321A and E323A in polδ inactivated its proofreading activity (30), and the L612M mutation in polδ decreased the fidelity of replication (31–33). We therefore used three promoters with different strengths to express *POL3^D321A^*^, ^*^E323A^*, *POL3^L612M^*, and *POL3^D321A^*^, ^*^E323A^*^, ^*^L612M^* and compared the mutation rate by measuring the mutation frequency in canavanine-resistant cells (see Table S1) (29). The mutation rate with the double point mutations D321A and E323A was higher than that with the single point mutation L612M and the triple point mutations D321A, E323A, and L612M (Table 1). Interestingly, the expression level of the *POL3* mutants did not significantly influence the mutation rate (see Table S1), which indicates that the expression of the *POL3* mutants using a weak promoter is sufficient for error-prone replication. Compared to the control strain, the expression of *POL3^D321A^*^,^*^ E323A^* increased the mutation rate ∼50-fold, reaching 7.5 × 10^−6^ (Table 1). This mutation rate is still lower than that in E. coli, in which the mutation rate increased by 10^2^- to 10^3^-fold with the expression of an error-prone DNA polymerase.

**TABLE 1:** Mutation rates of canavanine-resistant mutants in the mutator strains

Strain	Canavanine resistance mutation frequency^*a*^ (×10^−7^) ± SD	Fold elevation^*b*^
Control	4.2 ± 0.3	1
*ADH1p-POL3^L612M^*	75.1 ± 7.5	18.0
*ADH1p-POL3^D321A^* ^,^ *^ E323A^*	227.7 ± 16.7	54.4
*ADH1p-POL3^D321A^* ^,^ *^ E323A^* ^, ^ *^L612M^*	38.2 ± 5.6	9.1
*ΔPMS1*	314.3 ± 32.7	78.6
*ΔPMS1-ADH1p -POL3^D321A^* ^,^ *^ E323A^*	828.6 ± 63.5	198.8

a Means of three independent replications are shown.

b The fold elevation is the stain with *POL3* variant or ΔPMS1 relative to the stain with empty vector.

10.1128/msystems.01366-21.8 TABLE S1 Mutation rates of canavanine-resistant mutants in the mutator strains. Download Table S1, DOCX file, 0.02 MB. Copyright © 2022 Qiu et al. 2022 Qiu et al. https://creativecommons.org/licenses/by/4.0/ This content is distributed under the terms of the Creative Commons Attribution 4.0 International license.

To further elevate the mutation rate, we attempted to deleted the *PMS1* gene, which encodes an ATP-binding protein that participates in the DNA mismatch repair system (34). Indeed, the mutation rate in the *ΔPMS1* strain significantly increased the mutation rate about 80-fold (Table 1). When combining the *ΔPMS1* deletion with the error-prone *ADH1p-POL3^D321A-E323A^* strain, the mutation rate reached 8.3 × 10^−5^, an increase of 2 orders of magnitude. To confirm the reliability of the mutation rate calculation, we sequenced the *CAN1* gene in eight independent canavanine-resistant mutant strains generated from the *ΔPMS1-ADH1p-POL3^D321A-E323A^* strain. Each mutant had at least one base change within the *CAN1* coding sequence, and both transitions and transversions were observed (see Table S2). Therefore, we developed an *in vivo* mutagenesis system in S. cerevisiae that can improve the mutation rate by 10^2^-fold.

10.1128/msystems.01366-21.9 TABLE S2 Mutations in the *CAN1* gene in the *ΔPMS1-ADH1* derivative of CEN.PK-102-1C. Download Table S2, DOCX file, 0.01 MB. Copyright © 2022 Qiu et al. 2022 Qiu et al. https://creativecommons.org/licenses/by/4.0/ This content is distributed under the terms of the Creative Commons Attribution 4.0 International license.

### Screening for mutant strains with improved malonyl-CoA flux.

Next, we created a mutagenesis library in the *ΔPMS1-ADH1p-POL3^D321A-E323A^* strain by subculturing the cells and screening for mutant strains with an improved malonyl-CoA level using the growth-coupled circuit we developed. According to the mutation rate, the cells are required to replicate at least 13 generations to generate a genome-scale mutagenesis library. Five independent cultivations were performed, and each was subcultured twice when cells were grown to the late exponential phase. The cells were then collected and spread on plates containing 800 mg/L G418. To ensure the screening accuracy, two rounds of screening were performed. Both the *KanMX* and the *GFP* genes were expressed under the control of the malonyl-CoA biosensor to provide a cross validation for growth-based selection and fluorescence determination.

**FIG 3:**
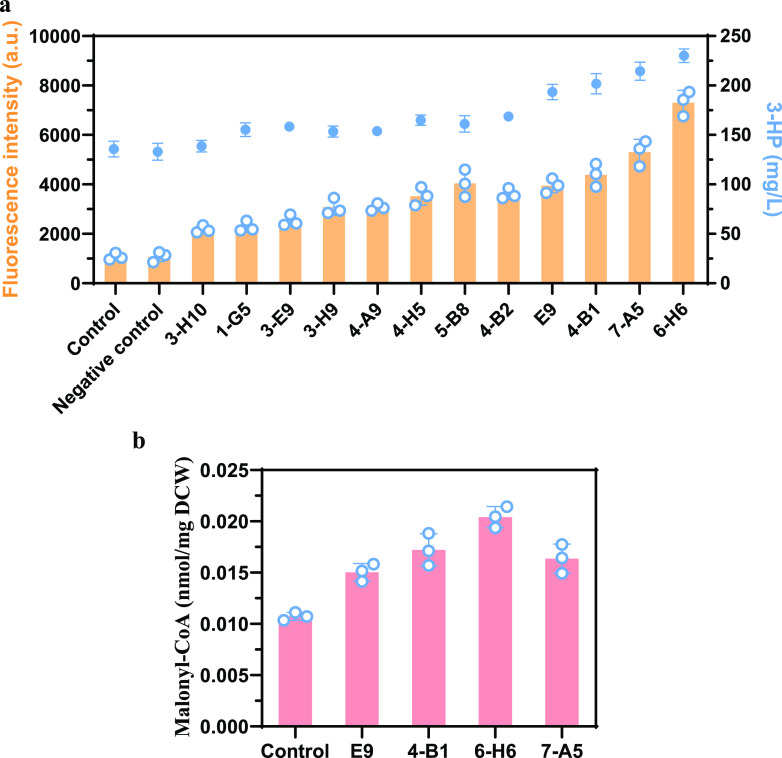
Selection of the mutant strains with enhanced malonyl-CoA flux. (a) Fluorescence intensities and 3-HP production profiles of 12 mutant strains from the G418-resistant plates and second round of fluorescence examination. (b) Intracellular malonyl-CoA concentration of the top four mutant strains with the highest 3-HP production. The control was CEN.PK2-1C. The negative control consisted of randomly picked colonies of mutation strains with non-high fluorescence and non-high-3-HP production. Error bars represent the means ± the standard deviations of three independent experiments.

About 200 colonies with better growth were selected from the plates containing 800 mg/L G418, and the fluorescence was then examined; 39 colonies with the highest fluorescence were selected for further analysis. To exclude the possibility that the mutation occurred in the biosensor containing plasmid and to prevent further unwanted mutations continued arising in the selected strains, we removed both the biosensor containing plasmid and the *ADH1p-POL3^D321A-E323A^* containing plasmid, and then reintroduced the malonyl-CoA biosensor plasmid containing the *GFP* expression cassette to these 39 strains. Of these strains, 12 of 39 showed an increased fluorescence level compared to the control in the second round of fluorescence examination (Fig. 3a). The 3-HP expression plasmid was then transformed into these 12 strains to determine the production level of malonyl-CoA downstream products. As shown in Fig. 3a, the titer of 3-HP indeed increased in most of the strains. The top four strains with the highest 3-HP production were selected for further analysis. The intracellular malonyl-CoA concentration was measured and consistent with 3-HP production, it was higher in these four mutant strains than control strain (Fig. 3b). The physiological characteristics of these strains were analyzed (see Fig. S3). Compared to the control strain, the strains with increased 3-HP production had a slight lower growth rate. The strain with the highest 3-HP titer (6-H6) had the lowest growth rate. The glucose consumption rates in the mutant strains were also slightly lower than those in the control, which indicates that the mutations affected the pathways related to growth and led to an increased malonyl-CoA flux.

10.1128/msystems.01366-21.3 FIG S3 The physical characterization of four mutant strains. (a to d) The growth (a), glucose consumption (b), ethanol production (c), and glycerol production (d) of four mutant strains in a shaking flask. The control is CEN.PK2-1C. Error bars represent the means ± the standard deviations from three independent experiments. Download FIG S3, EPS file, 1.3 MB. Copyright © 2022 Qiu et al. 2022 Qiu et al. https://creativecommons.org/licenses/by/4.0/ This content is distributed under the terms of the Creative Commons Attribution 4.0 International license.

### Whole-genome sequencing of the mutant strains and identification of the causal genes that affect the malonyl-CoA flux.

To analyze the characteristics of the *in vivo* mutagenesis and investigate the possible genes that affect malonyl-CoA synthesis, we performed whole-genome sequencing of these four mutant strains with improved 3-HP titer and on the original strain, CEN.PK2-1C. The number of nonsynonymous single nucleotide polymorphisms (SNPs) in coding region and insertions/deletions (InDels) in the four mutant strains is shown in Fig. S4a. We compared the proportion of mutations among the four mutant strains (see Fig. S4b) and found that SNPs were about 70% and InDels were about 30%. Among the InDels, the proportion of deletions was ∼2-fold higher than that of insertions. Among SNPs, the transitions, including the AT-to-GC substitution and the GC-to-AT substitution, accounted for 73% of all point mutations (see Fig. S4c), and the transversions accounted for 27% of all point mutations. Interestingly, our mutagenesis method generated a higher proportion of InDels and transversions than that generated by ethyl methanesulfonate (29) and UV mutagenesis (35).

10.1128/msystems.01366-21.4 FIG S4 Overview of genomic sequencing. (a) Summary of the numbers of genes containing SNPs and InDels in four mutant strains. (b) Classification of mutation type of four mutant strains. (c) Classification of single nucleotide substitutions of four mutant strains. Download FIG S4, EPS file, 1.3 MB. Copyright © 2022 Qiu et al. 2022 Qiu et al. https://creativecommons.org/licenses/by/4.0/ This content is distributed under the terms of the Creative Commons Attribution 4.0 International license.

Because the *in vivo* evolution generated hundreds of SNPs and InDels in each strain, we first analyzed the common changed genes in the mutant strains to identify the key targets. We then used GO Slim Process categories to cluster the SNP and InDel affected genes and analyzed the top 10 GO process category. Finally, genome sequencing was combined with transcriptome analysis to identify the causal genes related to the malonyl-CoA flux.

**FIG 4:**
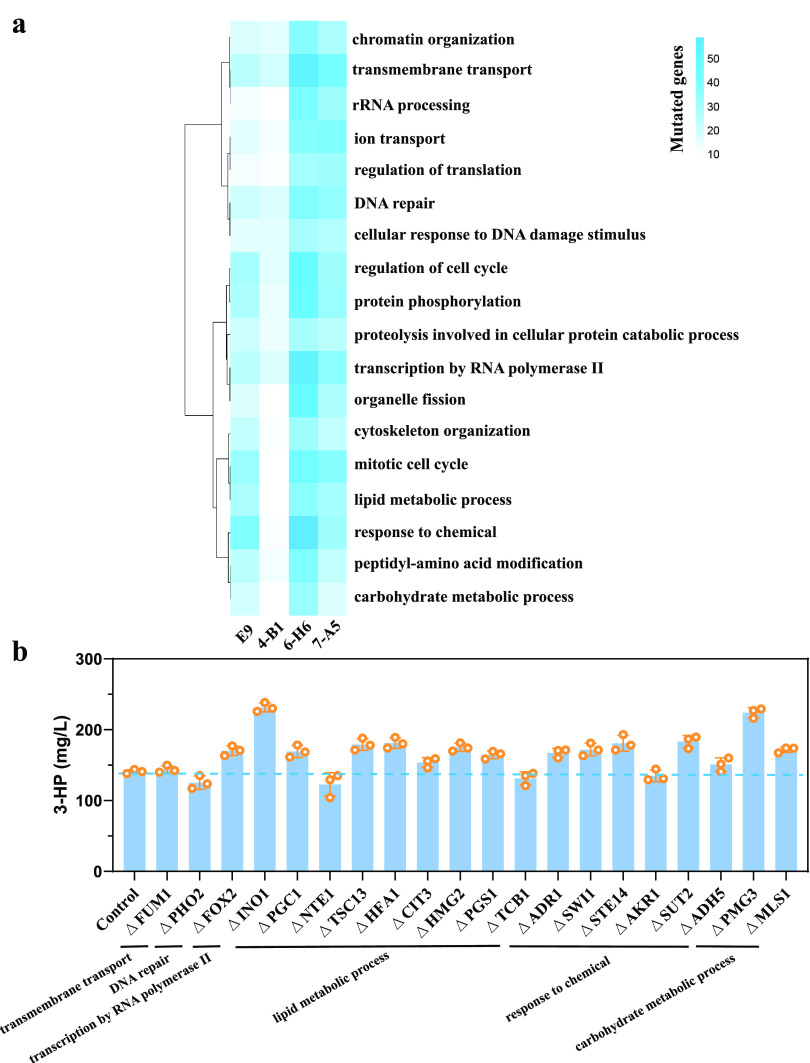
Identification of the key genes affected 3-HP production using GO Slim Process categories. (a) The top 10 GO terms of the mutated genes with InDels in each mutant strain as revealed by GO Slim Process categories. (b) 3-HP production of the strain deleting the genes with InDels that participate in transmembrane transport, DNA repair, transcription by RNA polymerase II, lipid metabolic process, and response to chemical and carbohydrate metabolic process. Error bars represent the means ± the standard deviations of three independent experiments.

To identify the common changed genes, the mutated genes in these four mutants were plotted in a Venn diagram (see Fig. S5a). The mutated genes involved in the central carbon metabolic pathway, lipid metabolism, transmembrane transport, and transcription regulation in at least two strains are summarized in Fig. S5b. Inverse engineering was then performed. We performed *in situ* site-directed mutations for the genes with SNPs and deleted the genes with InDels in the control strain and then determined the 3-HP production. The mutations that increased the 3-HP titer by >50% were considered causal mutations. However, the deletion of the genes with InDels and *in situ* site-directed mutations for the genes with SNPs did not significantly increase 3-HP production (see Fig. S5c and d). Although the *PGM2* mutation increased production by 27%, the change is not very significant. We therefore used the Gene Ontology (GO) Slim Mapper for the GO Slim Process analysis of the InDels and SNPs. Figure 4a shows the top 10 enriched GO categories in each mutated strain. We found that processes such as lipid metabolic process, ion transport, protein phosphorylation, and carbohydrate metabolic process that are related to metabolism were enriched in these four mutants. Based on this analysis, 20 genes with InDels in the categories related to carbon metabolism, including the lipid metabolic process and the carbohydrate metabolic process, were selected, and the 3-HP production was examined. We found that deletion of *INO1* or *PGM3* increased 3-HP production by >50% (Fig. 4b). Pgm3 catalyzes the interconversion of ribose-1-phosphate and ribose-5-phosphate and of glucose-1-phosphate and glucose-6-phosphate. Ino1 is an inositol-3-phosphate synthase involved in the synthesis of inositol phosphates and inositol-containing phospholipids. Pgm3 and Ino1 were reported to affect glycogen accumulation. Therefore, we determined the glycogen level. We found that *PGM3* deletion decreased glycogen accumulation, whereas *INO1* deletion did not significantly change it (see Fig. S5e). It seems that the regulation of storage carbohydrates by *PGM3* deletion affected the carbon flux to central carbon metabolism and improved malonyl-CoA synthesis.

10.1128/msystems.01366-21.5 FIG S5 Genome sequencing analysis and identification of causal mutations in the mutant strains with enhanced malonyl-CoA flux. (a) Venn diagram of the common mutated genes in four mutant strains. (b) Mutations present in at least two mutant strains and involved in central carbon metabolic pathway, lipid metabolism, transmembrane transport, or transcription regulation. (c) 3-HP production of the strains deleting the genes with InDels. (d) 3-HP production of the strains with *in situ* site-directed mutations in the genome. (e) The glycogen content of ΔPGM3, ΔINO1, and the control strain in exponential phase. The control strain is CEN.PK2-1C. Error bars represent the means ± the standard deviations from three independent experiments. Download FIG S5, EPS file, 1.4 MB. Copyright © 2022 Qiu et al. 2022 Qiu et al. https://creativecommons.org/licenses/by/4.0/ This content is distributed under the terms of the Creative Commons Attribution 4.0 International license.

### Identification of causal genes by transcriptome analysis.

**FIG 5:**
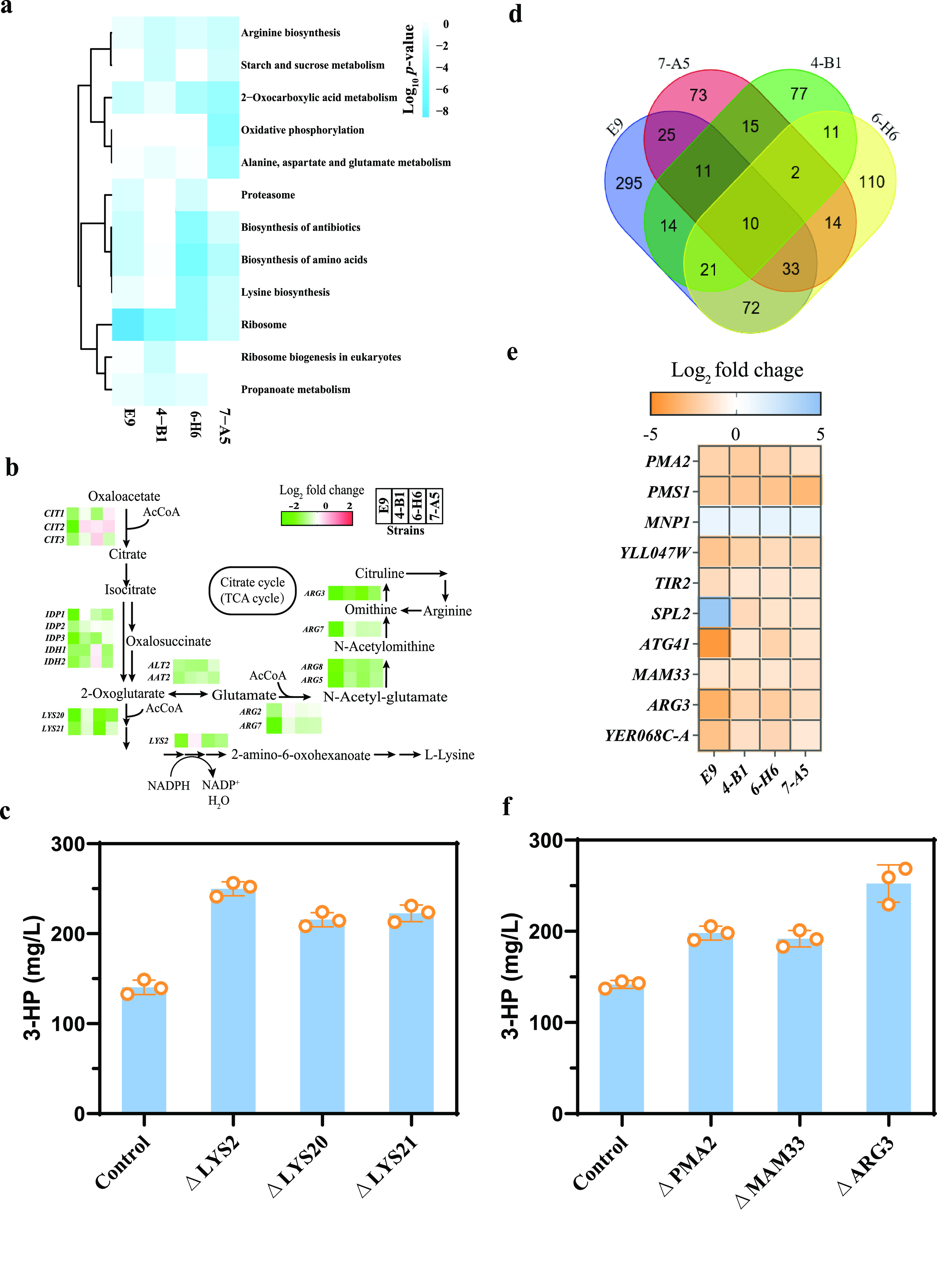
Transcriptome analysis of four mutant strains and the control strain and identification of causal mutations. (a) Top five KEGG enrichment pathways in four mutant strains. (b) Transcription levels of genes related to 2-oxocarboxylic acid metabolism and arginine and lysine biosynthesis in all four mutant strains. (c) 3-HP production in reverse engineered strains (*ΔLYS2*, *ΔLYS20*, and *ΔLYS21*). (d) Venn diagram of common significantly regulated genes in four mutant strains compared to the control strain, CEN.PK2-1C [*P* < 0.05 and abs (log_2_-fold change) > 1]. (e) Transcription levels of common significantly differentially expressed genes in all mutant strains. (f) 3-HP production in the reverse engineered strains (*ΔPMA2*, *ΔMAM33*, *and ΔARG3*) in which the transcription levels were significantly downregulated. Error bars represent the means ± the standard deviations of three independent experiments.

To further understand the changes in the mutant strains, we performed RNA-seq (transcriptome sequencing) and compared the transcriptome of the four mutants to that of the control strain. The numbers of genes significantly (*P* < 0.05) upregulated and downregulated and their mean expression levels are shown in Fig. S6a. The top five KEGG pathways revealed by KEGG enrichment analysis are presented in Fig. 5a. Interestingly, we found that, aside from ribosome synthesis, the biosynthesis of amino acids, such as arginine and lysine, as well as alanine, aspartate, glutamate, and 2-oxocarboxylic acid metabolism, was enriched in these four strains. We found that Gcn4, an amino acid regulator, was mutated in 6-H6, which may affect the transcription level of amino acid synthesis genes. This can also be reflected by KEGG enrichment pathway of 6-H6 (see Fig. S6b). Both arginine and lysine are derived from 2-oxocarboxylic acid metabolism. The transcription level of the genes in 2-oxocarboxylic acid metabolism, arginine biosynthesis, and lysine biosynthesis were significantly downregulated in all four mutant strains (Fig. 5b). To investigate whether this is related to malonyl-CoA availability, we searched for possible targets for inverse engineering. First, we identified the genes that had SNPs or InDels, and their transcription was significantly changed in the transcriptome profiles of these pathways. The *LYS2*, *LYS20*, and *LYS21* genes in the lysine biosynthesis pathway, which is also related to 2-oxoglutarate metabolism, were isolated. We then evaluated the effect of deleting *LYS2*, *LYS20*, and *LYS21* and found that indeed they resulted in increased 3-HP production (Fig. 5c). In particular, the deletion of the *LYS2* gene increased the 3-HP titer by ∼78%.

10.1128/msystems.01366-21.6 FIG S6 Transcriptome analysis and identification of causal mutations in the mutant strains with enhanced malonyl-CoA flux. (a) Summary of the numbers and mean expression levels of differentially expressed genes in four mutant strains compared to the control strain CEN.PK2-1C (*P* < 0.05). The mean value indicates the average of absolute expression level changes of up- and downregulated genes. (b) Top 14 KEGG pathways revealed by KEGG enrichment analysis for the mutant strain 6-H6 (*P* < 0.05). (c) 3-HP production of overexpressing *SDH3*, *BIO2*, *CBR1*, *PLB3*, and *DLD1* strains. Download FIG S6, EPS file, 1.5 MB. Copyright © 2022 Qiu et al. 2022 Qiu et al. https://creativecommons.org/licenses/by/4.0/ This content is distributed under the terms of the Creative Commons Attribution 4.0 International license.

Furthermore, we focused on the common changed genes in these four strains and plotted the genes with a log_2_-fold change of >1 or <−1 (Fig. 5d). There were 10 significantly changed genes in all mutated strains, and of these genes (Fig. 5e), *PMA2*, *MAM33*, and *ARG3* were deleted to check the production of 3-HP (Fig. 5f). The deletion of *ARG3* improved the 3-HP titer by 81%. Arg3 catalyzes the biosynthesis of the arginine precursor, citrulline, in the cytoplasm. In arginine biosynthesis, we found that like *ARG3*, the genes involved in catalyzing the formation of arginine from 2-oxoglutarate, such as *ARG2*, *ARG7*, *ARG5*, and *ARG8*, were also downregulated in the mutant strains.

Of the four deleted genes, *LYS2* or *ARG3* deletion decreased the cell growth slightly and caused lysine or arginine auxotrophy, respectively, whereas *LYS20* and *LYS21* deletion did not affect cell growth or cause auxotrophy (see Fig. S7a and d). The glucose uptake rate and ethanol production rate were also consistent with the growth (see Fig. S7b and c). We found that deletion of *LYS2* or *ARG3* blocked lysine and arginine synthesis completely, whereas deletion of *LYS20* and *LYS21* only downregulated these pathways. Interestingly, the increased level of 3-HP production was higher in *ΔLYS2* or *ΔARG3* strains than in *ΔLYS20* and *ΔLYS21* strains. It seems that downregulation of lysine and arginine synthesis correlated with the increased level of 3-HP. Amino acid synthesis is a carbon- and energy-consuming process. In the lysine and arginine synthesis process, acetyl-CoA is required, and the downregulation of these pathways reduced the consumption of acetyl-CoA, thereby increasing malonyl-CoA availability.

10.1128/msystems.01366-21.7 FIG S7 Physical characterization of strains downregulating lysine and arginine synthesis. (a to c) The growth (a), glucose consumption (b), and ethanol production (c) of ΔLYS2, ΔLYS20, ΔLYS21, and ΔARG3 strains and the control strain in a medium containing 20 amino acids. (d) Spot assay of ΔLYS2, ΔLYS20, ΔLYS21, and ΔARG3 strains and the control strain in medium without arginine and lysine. Error bars represent the means ± the standard deviations from three independent experiments. Download FIG S7, PDF file, 0.6 MB. Copyright © 2022 Qiu et al. 2022 Qiu et al. https://creativecommons.org/licenses/by/4.0/ This content is distributed under the terms of the Creative Commons Attribution 4.0 International license.

In addition to the downregulated genes, several upregulated genes were identified from the common changed genes, including *SDH3*, *BIO2*, *CBR1*, *PLB3*, and *DLD1*. We found that overexpression of *SDH3* and *BIO2* improved 3-HP production (see Fig. S6c). The succinate dehydrogenase subunit (Sdh3) catalyzes the oxidation of succinate to fumarate and may promote energy metabolism. Bio2 is a biotin synthase and catalyzes the conversion of desulfurized biotin to biotin, which is the last step of the biotin biosynthesis pathway. Biotin is required for acetyl-CoA carboxylase (AccI), which is the main enzyme to produce cytosolic malonyl-CoA.

In addition to identifying common changed genes in all the mutant strains, we analyzed the transcription change in the mutant strain, 6-H6, which had the highest 3-HP yield and a slightly lower growth rate. We found that fatty acid synthesis was changed significantly in the KEGG pathway of this strain (see Fig. S6b). Fatty acid synthesis is the major source malonyl-CoA consumption, and its regulation can directly affect the malonyl-CoA availability. The downregulation of this pathway may reduce malonyl-CoA consumption and increase 3-HP production at the expense of cell growth (36).

## DISCUSSION

Here, we developed a growth-based genetic system that coupled an intracellular metabolite concentration to cell growth to evolve strains with improved metabolite synthesis. Growth-based screening is generally limited to evolve phenotypes such as stress tolerance and substrate utilization. It is often difficult to screen for a phenotype with improved metabolite production. When linking cell growth with metabolite concentration, the beneficial mutations can be easily enriched without requiring expensive equipment such as are needed for FACS and droplet-based microfluidics cell sorting. However, only a few studies have created such a system in yeast. For example, Leavitt et al. have reported that an aromatic amino acid biosensor was coupled with an antimetabolite selection scheme to screen for improved muconic acid production (9). Malonyl-CoA is an intracellular metabolite, and in our study, as a proof-of-concept, we established a growth-based screening system that couples malonyl-CoA to either a toxicity gene or an antibiotic resistance gene. Some growth-coupled systems create an auxotrophic phenotype to couple the metabolite biosynthesis with cell growth (37). However, the design is often pathway specific and cannot be transferred to other metabolites. In addition, after the mutant screening, it may be necessary to complement the auxotrophy for the subsequent application. Conversely, the antibiotic resistance gene can be coupled to many transcription factor-based biosensors to create a growth-based system. Therefore, our design principle can be easily transferred to other metabolites for rapid screening for high producers. The limitation of this system is that the expression level of *KanMX* does not linearly correlate with the cell growth. Namely, once a certain amount of KanMX is produced, it can recover the growth in of 800 mg/L G418. Therefore, we did not perform continuous evolution and isolate the mutant with the even higher malonyl-CoA availability. In future work, a degradation tag will be fused to *KanMX* to expand the screening range.

Mutagenesis is a powerful tool for generating genetic diversity and has been applied extensively to evolve industrial microorganisms. The commonly used mutagenesis strategies, such as physical and chemical mutagenesis (15), can easily generate mutation libraries but cannot achieve continuous mutations. *In vivo* mutagenesis can accelerate strain evolution through introducing genetic perturbations into the genome replication machinery. This method allows continuous mutagenesis *in vivo* by simple serial subculturing. When coupled with growth-based selection, it is possible to achieve continuous mutagenesis and selection simultaneously, which cannot be achieved by other high-throughput screening methods.

**FIG 6:**
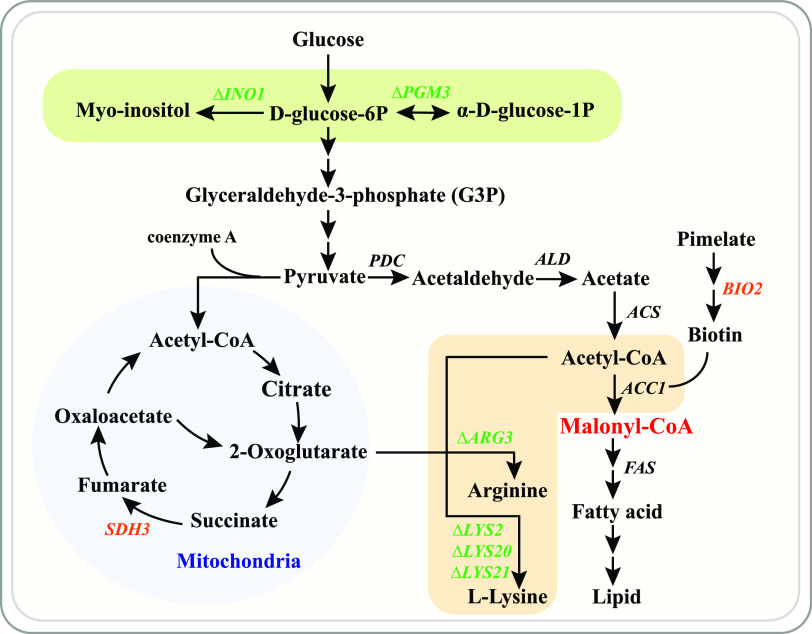
Summary of genes that improved the malonyl-CoA flux. The genes in orange represent overexpressed genes, and the genes in green represent deleted genes.

Coupling *in vivo* mutagenesis with growth-based screening enabled screening of the strains with improved malonyl-CoA synthesis. Since the strains possess many genomic mutations causing changes in many different intracellular processes, it would be difficult to identify the effects of all these mutations. However, from our systems level analysis we could identify common regulation patterns and thereby specified the general rules required for improved malonyl-CoA flux (Fig. 6). Using omics analysis, we identified new targets that can enhance malonyl-CoA availability and 3-HP production. They mainly increase acetyl-CoA or malonyl-CoA flux through either eliminating the carbon flux to other metabolic pathways, such as the flux to carbohydrate storage, and synthesis of arginine or lysine, or increasing the acetyl-CoA or malonyl-CoA synthesis.

It is reported that optimizing the supply of acetyl-CoA and malonyl-CoA can increase the production of 3-HP (8). Unlike these previously reported targets that mainly increase malonyl-CoA by improving its synthesis, some of the new targets we identified here affect malonyl-CoA availability through regulating the carbon flux to other metabolic pathways. Importantly, we revealed for the first time that weakening the amino acid synthesis pathway has a positive effect on increasing malonyl-CoA. Amino acids are building blocks for protein synthesis, and their synthesis is a carbon- and energy-consuming process (38). Downregulation of lysine and arginine synthesis genes may therefore allocate more carbon for malonyl-CoA production. Arginine and lysine synthesis requires acetyl-CoA; hence, downregulation of these pathways will save the carbon for the synthesis of desired products. Moreover, suppressing the lysine and arginine pathways did not cause significant growth inhibition, which indicates the redundancy of the enzymes of these pathways. Even though the deletion of *LYS2* or *ARG3* caused auxotrophy in the presence of amino acids in the medium, the growth difference was small.

In summary, we developed a new growth-based screening approach that couples an *in vivo* mutagenesis system with a growth-based genetic circuit for screening mutant strains with improved malonyl-CoA availability in S. cerevisiae. The approach developed here coupled the metabolite concentration to a growth-based phenotype in yeast and will be a valuable complementary tool to the existing method for high-throughput screening. Based on the omics analysis of the mutant strains, we identified new processes such as altered carbohydrate storage and reduced lysine and arginine synthesis that enhanced malonyl-CoA availability and 3-HP production. These new targets will help to elucidate the cellular regulations associated with malonyl-CoA metabolism and the construction of efficient cell factories for diverse chemical production.

## MATERIALS AND METHODS

### Media and culture conditions.

Escherichia coli strain DH5α was used for plasmid isolation and was cultivated in Luria-Bertani (LB) broth (5 g/L yeast extract, 10 g/L tryptone, and 10 g/L NaCl) at 37°C with 200-rpm orbital shaking. The LB medium was supplemented with 100 mg/L ampicillin for plasmid maintenance and propagation (39).

YPD medium containing 10 g/L yeast extract and 20 g/L peptone, supplemented with 20 g/L glucose, was used to culture yeast cells without any plasmid. Synthetic complete (SC) dropout medium was used to cultivate the S. cerevisiae strains. The medium comprised 1.7 g/L yeast nitrogen base (BBI Life Science Corporation, China), 5 g/L ammonium sulfate, SC dropout medium without uracil, leucine, and/or histidine (Sunrise Science Products, San Diego, CA), and 20 g/L glucose. When necessary, 800 mg/L G418 (Promega Corporation, Madison, WI), 60 mg/L l-canavanine (Sigma-Aldrich, St. Louis, MO), or 5′-FOA (Sigma-Aldrich) was added to the growth medium. All S. cerevisiae strains were cultivated at 30°C with 200-rpm shaking. Yeast cells were precultured in 5 mL of medium in 40-mL Falcon tubes and transferred to 40 mL of medium for cultivation in 100-mL shake flasks in the manuscript.

### Strains and plasmids construction.

All strains and plasmids used and constructed in this study are listed in (see Table S3), respectively. Plasmids were constructed by restriction enzyme digestion and ligation or Gibson assembly.

10.1128/msystems.01366-21.10 TABLE S3 Plasmids and strains used in this study. Download Table S3, DOCX file, 0.02 MB. Copyright © 2022 Qiu et al. 2022 Qiu et al. https://creativecommons.org/licenses/by/4.0/ This content is distributed under the terms of the Creative Commons Attribution 4.0 International license.

S. cerevisiae CEN.PK2-1C (*MAT***a** *ura3-52 trp1-289 leu2-3*,*112 his3Δ1 MAL2-8C SUC2*) was used as the initial strain for strain engineering. To construct the *in vivo* mutagenesis system, nine combinations of *POL3^D321A^*^, ^*^E323A^*, *POL3^L612M^*, and *POL3^D321A^*^ ^*^E323A^*^, ^*^L612M^* regulated by *TEF1p*, *ADH1p*, and *CYC1p* were cloned into the single-copy plasmid, pJFE1. For constructing the growth-based screening circuits, the *KanMX* cassette was cloned in plasmid pJfapO-fapR instead of *yeGFP* (21). A codon-optimized malonyl-CoA reductase *MCR* gene from Chloroflexus aurantiacus was divided into two fragments. The N-terminal region of *MCR* (MCR-N; amino acids 1 to 549) under the control of the *CYC1* promoter and the C-terminal region of *MCR* (MCR-C; amino acids 550 to 1219) under the control of the *PGK1* promoter were ligated into the 2μ plasmid, pIYC04.

The genes with InDels and the downregulated genes were deleted using *KanMX* as a selection marker. The upregulated genes were amplified and expressed under the control of the *TEF1* promoter and the *CYC1* terminator in pJFE3. CRISPR was used for site-directed mutagenesis in the genome. The plasmid carrying the *Cas9* gene was first transformed into the yeast strain CEN.PK2-1C. Another plasmid carrying gRNAs for *UGP1*, *LAT1*, *GDB1*, and *PGM2* mutation was cotransformed with donor DNA fragments containing 120-bp flanking homologous sequences, including site-directed mutation of the target gene and synonymous mutation of PAM. The transformants were cultivated in the corresponding resistance plates, and the gene mutation was verified by colony PCR and sequencing.

### Mutation rate measurement.

The plasmids carrying the *POL3* variants (pJFE1 empty vector was used as a control) were introduced into S. cerevisiae CEN.PK2-1C and *ΔPMS1* strain cells. Yeast cells grown in SC-URA medium to the stationary phase were washed and suspended in water. Cells (OD_600_ = 1) were spread on SC-URA-ARG plates with or without 60 mg/L canavanine. The mutation rates were measured by calculating the number of canavanine-resistant colonies divided by the number of colonies on the SC-URA-ARG plate without canavanine.

### Fluorescence intensity measurement.

Fluorescence intensity was measured with a 1420 Multilabel Counter (Victor3 V; Perkin-Elmer, Waltham, MA). The cell density was measured at 600 nm (Eppendorf BioPhotometer, Germany), and the excitation and emission wavelengths for green fluorescent protein (GFP) were 485 ± 20 and 585 ± 20 nm, respectively. The fluorescence intensity (a.u.) was normalized to the cell density (OD_600_). To measure the fluorescence of the strains with different malonyl-CoA levels, an overnight culture was collected, inoculated into fresh SC medium with an initial OD_600_ of 0.2 and cultivated for 12 h. The fluorescence intensity was then measured. For mutant strains screening, strains were picked from resistance medium plate and inoculated into 200 μL of fresh SC medium in 96-well plates. The OD_600_ and fluorescence were detected using a Multi-Detection microplate reader (Synergy HT; BioTtek, Winooski, VT).

### Genome-scale mutagenesis library generation.

Briefly, the estimated mutation rate per base is 8.3 × 10^−5^ substitutions by statistical analysis. With genome size of yeast 12 Mb, we expected about 10^3^ mutations/replication/generation. Therefore, to create a mutation library that can cover the whole genome, the passage number was calculated using the following equation:

$$S=\frac{a_{1}(1\;-\;q^{n})}{1\;-\;q},$$

where *a*_1_ is 10^3^ mutations in the first generation, *S* represents the size of the whole genome, and *n* is the number of generations. According to the way that yeast cells divide, *q* is equal to 2. We calculated that the cells are required to propagate for more than 13 generations to obtain a mutation library covering all bases of the entire genome. Considering the doubling time of yeast in SC medium is ∼3 h, more than 40 h of cultivation should generate a genome-scale mutagenesis library. The *ΔPMS1-ADH1p-POL3^D321A-E323A^* strain was inoculated and cultivated in SC medium, and it was transferred to a fresh medium when the growth reached the mid-exponential phase. After 40 h, the culture was plated on a medium containing 800 mg/L G418. Five independent cultivations were performed.

### Glycogen assays.

The glycogen content was analyzed as described previously (40). Briefly, yeast cultures were grown to the midlog stage, and ∼100 OD_600_ of culture was harvested and washed twice with H_2_O. The pellets were collected and frozen in liquid nitrogen. The pellets were resuspended in 500 μL of 0.25 M Na_2_CO_3_, followed by incubation for 4 h at 95°C. Then, 0.15 mL of 1 M acetic acid and 0.65 mL of 0.2 M sodium acetate (pH 5.2) were added to the sample. Glycogen was digested by 70 U/mg amyloglucosidase (10115; Sigma-Aldrich) overnight at 57°C. After digestion, the supernatants were collected, and the glucose concentration in the supernatant was measured using a glucose colorimetric/fluorometric assay kit (MAK263; Sigma-Aldrich).

### Extraction and quantification of intracellular malonyl-CoA.

For malonyl-CoA quantification, S. cerevisiae cell were cultured in the midexponential phases, and 10 mL of culture broth was sampled rapidly into preweighed tubes containing 30 mL of prechilled methanol (−40°C) quenching solution (41). The sample were separated by centrifugation at 3,000 × *g* for 20 min at −20°C, resuspended in 2.5 mL of cold methanol and 1 mL of chloroform, and then stored at −80°C. The malonyl-CoA was extracted by adding 4 mL of chloroform (−20°C) and 2 mL of 3 mM PIPES-EDTA (pH 7), followed by vigorous shaking at 500 rpm for 45 min at −20°C (42). All samples were separated by centrifugation (3,000 × *g*, 20 min, −20°C), and the supernatant was collected for quantifying malonyl-CoA by using a malonyl-CoA enzyme-linked immunoassay (JL46963; Jianglaibio, China).

### Metabolite analysis.

Strains were precultured in tubes with 5 mL of SC medium. Seed cultures were inoculated into shake flasks with a working volume of 40 mL and an initial OD_600_ of 0.2 for 48 h. The concentrations of 3-HP, ethanol, glucose, and glycerol were measured by high-pressure liquid chromatography (HPLC; Shimadzu Corporation, Japan) equipped with an Aminex HPX-87H column (Bio-Rad, Hercules, CA). H_2_SO_4_ (2.5 mM) was used as the mobile phase with a flow rate of 0.6 mL/min, and the temperature of the column was 65°C.

### Whole-genome sequencing.

Yeast cultures were harvested in the midlog phase, and genomic DNA was extracted using a previously described method (SDS method) (43). Sequencing libraries were generated using a NEBNext Ultra DNA Library Prep kit according to manufacturer’s guidelines, and the DNA was fragmented to 350 bp. The samples were sequenced using Illumina NovaSeq PE150 at the Beijing Novogene Bioinformatics Technology Co., Ltd. (Beijing, China). The sequences were mapped to the reference genome of CEN.PK 113-7D using BWA software (version 0.7.8) (44) software and SAMTools (version 0.1.18) (45). The mapping results were used to identify SNPs and InDels between the mutant strains and the reference strain using Breakdancer (version 1.4.4) (46) software. GO Slim Term Mapper analysis was conducted with the online tool on the *Saccharomyces* Genome Database (SGD) website (http://www.yeastgenome.org/cgi-bin/GO/goSlimMapper.pl). Cluster analysis was also performed using the tools supplied on the website.

### Transcriptome analysis.

Yeast cells were cultured to the midexponential phase (OD_600_ ≈ 1), centrifuged, and frozen in liquid nitrogen. Total RNA was extracted by using a UNIQ-10 TRIzol RNA purification kit (Sangon Biotech, China). The RNA was processed by mRNA enrichment or rRNA removal. The RNA samples were sequenced by an Illumina HiSeq 2500 (performed by Beijing Genomics Institute) with paired ends (2 × 125 bp) according to the manufacturer’s instructions. The clean reads from each sample were mapped to the reference genome of CEN.PK113-7D (https://www.yeastgenome.org/strain/S000203459), with an average of 87.91% of the reads being successfully mapped. Analysis of the differentially expressed genes (DEGs) was carried out in R using the DEGs package. Significantly changed genes (*P* < 0.05) were identified. All annotations were derived from the SGD (http://www.yeastgenome.org/). All analyses were performed in biological triplicates.

### Data availability.

Whole-genome sequencing and RNA-seq raw data for the mutant engineered strains were deposited to the NCBI Sequence Read Archive under the BioProjects PRJNA750225 and PRJNA750524. The data that support the findings of this study are available within the article.
