# Supplementary material for: Biosensor-Coupled In Vivo Mutagenesis and Omics Analysis Reveals Reduced Lysine and Arginine Synthesis To Improve Malonyl-Coenzyme A Flux in Saccharomyces cerevisiae
Source: mSystems. 2022 Mar 1;7(2):e01366-21. doi: 10.1128/msystems.01366-21 (PMC9040634; doi:10.1128/msystems.01366-21)
Supplement: TABLE S1 [file msystems.01366-21-st001.docx]

**Table S1**

| Strains | Mutation frequency of  Canavanine resistant (×10^−7^) | Fold elevation^a^ |
| --- | --- | --- |
| control | 4.2 ± 0.3 | 1 |
| *TEF1p-POL3^D321A, E323A^* | 190.9 ± 14.1 | 47.5 |
| *TEF1p-POL3^L612M^* | 44.7 ± 2.5 | 10.8 |
| *TEF1p-POL^3D321A, E323A, L612M^* | 84.8 ± 6.4 | 20.2 |
| *CYC1p-POL3^D321A, E323A^* | 206.3 ± 22.9 | 49.3 |
| *CYC1p-POL3^L612M^* | 65.5 ± 3.8 | 15.6 |
| *CYC1p-POL3^D321A, E323A, L612M^* | 48.0 ± 5.8 | 11.5 |
| *ΔPMS1-CYC1p-POL3^D321A, E323A^* | 618.3 ± 16.7 | 147.8 |
| ^a^ Fold elevation is the stain with *POL3* variant relative to the stain with empty vector.  Mean ± standard deviation of three independent replications. | | |
