# Supplementary material for: Biosensor-Coupled In Vivo Mutagenesis and Omics Analysis Reveals Reduced Lysine and Arginine Synthesis To Improve Malonyl-Coenzyme A Flux in Saccharomyces cerevisiae
Source: mSystems. 2022 Mar 1;7(2):e01366-21. doi: 10.1128/msystems.01366-21 (PMC9040634; doi:10.1128/msystems.01366-21)
Supplement: TABLE S2 [file msystems.01366-21-st002.docx]

**Table S2**

| Class | Change | Occurrences | Mutation |
| --- | --- | --- | --- |
| Transitions | G·C → A·T | 9 | G123→A(8), G498→A(1) |
|  | A·T →G·C | 4 | A1600→G(4) |
| Transversions | G·C → C·G | 0 |  |
|  | G·C → T·A | 2 | G897→T(1), C452→A(1) |
|  | A·T → C·G | 3 | T465→G(3) |
|  | A·T → T·A | 1 | A1257→T(1) |
| Deletions | -1 | 2 | A174, T625 |
| Insertions | +1 | 1 | 1112+A |
| Others |  | 0 |  |
| Total |  | 22 |  |
