# Supplementary figures and images for: Biosensor-Coupled In Vivo Mutagenesis and Omics Analysis Reveals Reduced Lysine and Arginine Synthesis To Improve Malonyl-Coenzyme A Flux in Saccharomyces cerevisiae
Source: mSystems. 2022 Mar 1;7(2):e01366-21. doi: 10.1128/msystems.01366-21 (PMC9040634; doi:10.1128/msystems.01366-21)

**Figure S7**

**a**

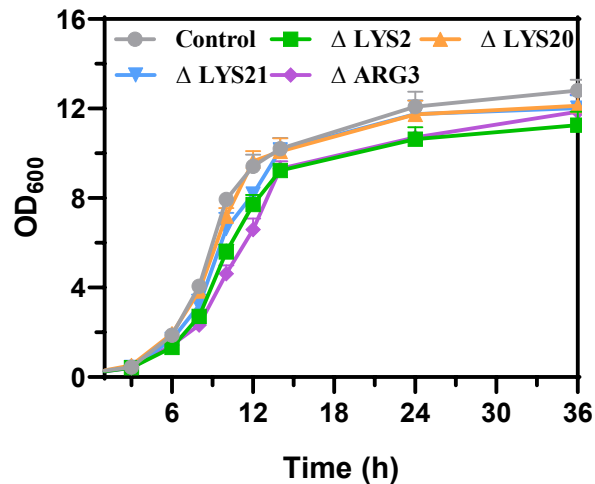

**b**

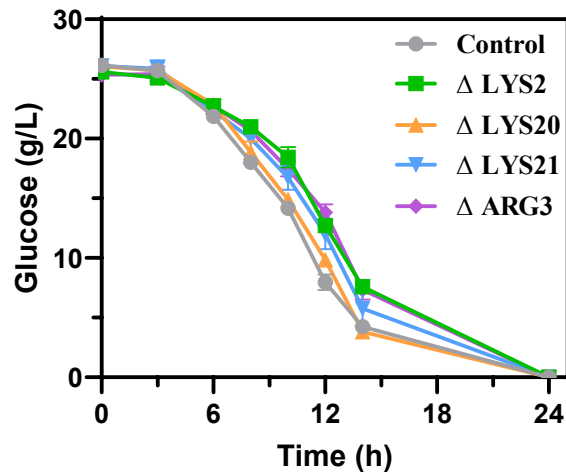

**c**

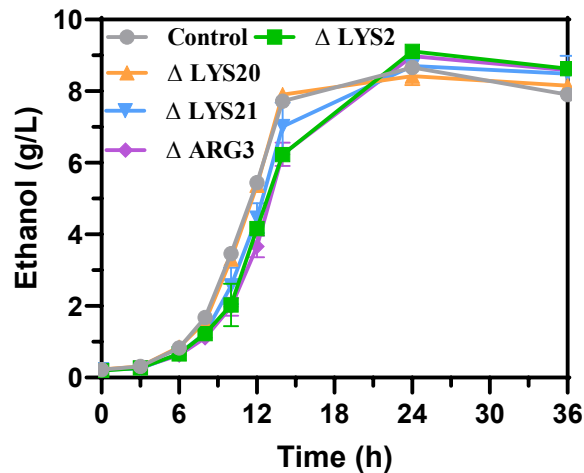

**d**

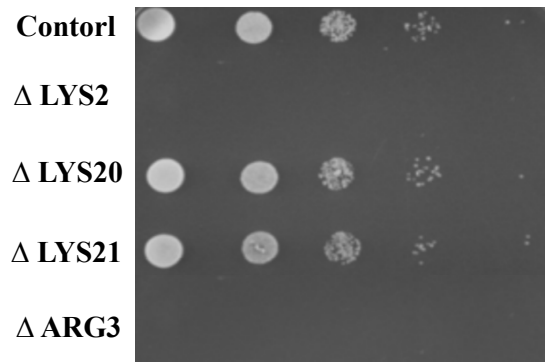

Supplement: FIG S7 [file msystems.01366-21-sf007.pdf]
