# Supplementary material for: Biosensor-Coupled In Vivo Mutagenesis and Omics Analysis Reveals Reduced Lysine and Arginine Synthesis To Improve Malonyl-Coenzyme A Flux in Saccharomyces cerevisiae
Source: mSystems. 2022 Mar 1;7(2):e01366-21. doi: 10.1128/msystems.01366-21 (PMC9040634; doi:10.1128/msystems.01366-21)
Supplement: TABLE S3 [file msystems.01366-21-st003.docx]

**Table S3**

| Plasmids | Description | Resource |
| --- | --- | --- |
| pJFE1 | CEN/ARS, amp^r^, *TEF1*p-*PGK1*t, *URA3* | Lab store |
| pJFE3 | 2µ, amp^r^, *TEF1*p-*PGK1t*, *URA3* | Lab store |
| pYX242-WS | 2µ, amp^r^, *TEF1*p-*polyA*, *TPI1*p-*PGK1t*, *LEU2* | Lab store |
| pIYC04 | 2µ ori, *TEF1p*-*ADH1t*, *PGK1p*-*CYC1t*, *HIS3* | (1) |
| pUG6 | ori, loxP-*KanMX*-loxP | (1) |
| PjTE-POL301 | CEN/ARS, amp^r^, *TEF1*p-*POL3^E321A-E323A^*-*PGK1*t, *URA3* | This study |
| PjTE-POL302 | CEN/ARS, amp^r^, *TEF1*p-*POL3^L612M^*-*PGK1*t, *URA3* | This study |
| PjTE-POL303 | CEN/ARS, amp^r^, *TEF1*p-*POL3 ^E321A-E323A -L612M^*-*PGK1*t, *URA3* | This study |
| PjAD-POL301 | CEN/ARS, amp^r^, *ADH1*p-*POL3^E321A-E323A^*-*PGK1*t, *URA3* | This study |
| PjAD-POL302 | CEN/ARS, amp^r^, *ADH1*p-*POL3^L612M^*-*PGK1*t, *URA3* | This study |
| PjAD-POL303 | CEN/ARS, amp^r^, *ADH1*p-*POL3 ^E321A-E323A -L612M^*-*PGK1*t, *URA3* | This study |
| PjCY-POL301 | CEN/ARS, amp^r^, *CYC1*p-*POL3^E321A-E323A^*-*PGK1*t, *URA3* | This study |
| PjCY-POL302 | CEN/ARS, amp^r^, *CYC1*p-*POL3^L612M^*-*PGK1*t, *URA3* | This study |
| PjCY-POL303 | CEN/ARS, amp^r^, *CYC1*p-*POL3 ^E321A-E323A -L612M^*-*PGK1*t, *URA3* | This study |
| pYlACL | 2µ ori, *TEF1p*-*YlACL2*-*ADH1t*, *PGK1p*-*YlACL1*-*CYC1t*, *HIS3* | This study |
| pJfapO-GFP-fapR | 2µ, amp^r^, *GAL1*(7)*fapO*p-*yeGFP-PGK1*t, *TEF1*p-*fapR*-*polyA*, *LEU2* | (2) |
| pRS304-01 | pRS304*-LEU2p·1*fapO-GFP-PGK1t* | (3) |
| pRS304-FCY1 | pRS304*-LEU2p·1*fapO-FCY1-PGK1t* | This study |
| pYFapR-M | pYX242-*TEF1p*-*FapR-Med2-PGK1t* | (3) |
| pJfapO-Kan^r^ | 2µ, amp^r^, *GAL1*(7)*fapO*p-*KanMX-PGK1*t | This study |
| pJfapO-fapR  -GFP-Kan^r^ | 2µ, amp^r^, *GAL1*(7)*fapO*p-*yeGFP-PGK1*t, *GAL1*(7)*fapO*p-*KanMX-PGK1*t, *TEF1*p-*fapR*-*polyA*, *LEU2* | This study |
| pJfapO-fapR-Kan^r^ | 2µ, amp^r^, *GAL1*(7)*fapO*p-*KanMX-PGK1*t, *TEF1*p-*fapR*-*polyA*, *LEU2* | This study |
| p-Cas9 | f1 ori, NAT ^r^, *TEF1*p -Cas9-*CYC1t* | (4) |
| pSH42H-gRNA | PSH42, 2µ, ori, hygB^r^, *SNR52p*-gRNA-*SUP4t* | This study |
| pJFE3-BIO2 | 2µ, amp^r^, *TEF1*p-*BIO2*-*PGK1t*, *URA3* | This study |
| pJFE3-SDH3 | 2µ, amp^r^, *TEF1*p-*SDH3*-*PGK1t*, *URA3* | This study |
| pJFE3-CBR1 | 2µ, amp^r^, *TEF1*p-*CBR1*-*PGK1t*, *URA3* | This study |
| pJFE3-DLD1 | 2µ, amp^r^, *TEF1*p-*DLD1*-*PGK1t*, *URA3* | This study |
| pJFE3-PLB3 | 2µ, amp^r^, *TEF1*p-*PLB3*-*PGK1t*, *URA3* | This study |
| pIYC04-Mcr | 2µ ori, *CYC1p-MCRN-ADH1t*, *PGK1p*-*MCRC-CYC1t*, *HIS3* | This study |
| pJFE3-Mcr | 2µ, amp^r^, *TEF1*p-*Mcr*-*PGK1t*, *URA3* | This study |

| Strains | Genotype | Source |
| --- | --- | --- |
| CEN.PK2-1C | *MATa; ura3-52; trp1-289; leu2-3,112; his3Δ1; MAL2-8C; SUC2* | This study |
| *ΔICL1* | CEN.PK2-1C; *ΔICL1::loxP* | This study |
| ACL01 | CEN.PK2-1C; *ΔICL1::loxP;* Detal15::*loxp*-P*_TEF1_*-YHM2-T*_ADH1_*, pYlACL | This study |
| ACL02 | CEN.PK2-1C; *ΔICL1::loxP;* Detal15::*loxp*-P*_TEF1_*-CTP1-T*_ADH1_*  -P*_PGK1_*-OAC1-T*_CYC1_*, pYlACL | This study |
| ACL03 | *ΔICL1*; pJFE3, pIYC04 | This study |
| ACL04 | ACL01; pJfpO-GFP-fapR | This study |
| ACL05 | ACL02; pJfpO-GFP-fapR | This study |
| ACL06 | ACL01; pJfapO-fapR-Kan^r^ | This study |
| ACL07 | ACL02; pJfapO-fapR-Kan^r^ | This study |
| ACL08 | ACL01; pJFE3-Mcr | This study |
| ACL09 | ACL02; pJFE3-Mcr | This study |
| QCse36 | CEN.PK2-1C; *trp1::1*fapO·LEU2p-FCY1-PGK1t* | This study |
| QCse37 | CEN.PK2-1C; *trp1::1*fapO·LEU2p-FCY1-PGK1t*, pYFapR-M | This study |
| QCse38 | *CEN.PK2-1C;* pJfapO-fapR-Kan^r^ | This study |
| QCse39 | *CEN.PK2-1C;* pJfapO-Kan^r^ | This study |
| ΔPMS1 | CEN.PK2-1C; *ΔPMS1::loxP* | This study |
| POL301 | CEN.PK2-1C; PjTE-POL301 | This study |
| POL302 | CEN.PK2-1C; PjTE-POL302 | This study |
| POL303 | CEN.PK2-1C; PjTE-POL303 | This study |
| POL304 | CEN.PK2-1C; PjAD-POL301 | This study |
| POL305 | CEN.PK2-1C; PjAD-POL302 | This study |
| POL306 | CEN.PK2-1C; PjAD-POL303 | This study |
| POL307 | CEN.PK2-1C; PjCY-POL301 | This study |
| POL308 | CEN.PK2-1C; PjCY-POL302 | This study |
| POL309 | CEN.PK2-1C; PjCY-POL303 | This study |
| POL3010 | ΔPMS1; PjCY-POL301 | This study |
| POL3011 | ΔPMS1; PjAD-POL301 | This study |
| 6-H6 | Evolved CEN.PK2-1C | This study |
| 7-A5 | Evolved CEN.PK2-1C | This study |
| E9 | Evolved CEN.PK2-1C | This study |
| BIO2 | CEN.PK2-1C; pJFE3-BIO2 | This study |
| SDH3 | CEN.PK2-1C; pJFE3-SDH3 | This study |
| CBR1 | CEN.PK2-1C; pJFE3-CBR1 | This study |
| DLD1 | CEN.PK2-1C; pJFE3-DLD1 | This study |
| LAT1^T457A^ | CEN.PK2-1C(*LAT1^T457A^*) | This study |
| GDB1^I947T^ | CEN.PK2-1C(*GDB1^I947T^*) | This study |
| UGP1^I484M^ | CEN.PK2-1C(*UGP1^I484M^*) | This study |
| UGP1^P386S^ | CEN.PK2-1C(*UGP1^P386S^*) | This study |
| PGM2^A288V^ | CEN.PK2-1C(*PGM2^A288V^*) | This study |
| PGM2^R586C^ | CEN.PK2-1C(*PGM2^R586C^*) | This study |
| ΔACE2 | CEN.PK2-1C; *ΔACE2::loxP* | This study |
| ΔCYC8 | CEN.PK2-1C; *ΔCYC8::loxP* | This study |
| ΔSDH4 | *CEN.PK2-1C*; *ΔSDH4::loxP* | This study |
| ΔTCB1 | CEN.PK2-1C; *ΔTCB1::loxP* | This study |
| ΔLYP1 | CEN.PK2-1C; *ΔLYP1::loxP* | This study |
| ΔPSK1 | CEN.PK2-1C; *ΔPSK1::loxP* | This study |
| ΔNTE1 | CEN.PK2-1C; *ΔNTE1::loxP* | This study |
| ΔHFA1 | CEN.PK2-1C; *ΔHFA1::loxP* | This study |
| ΔPGM3 | CEN.PK2-1C; *ΔPGM3::loxP* | This study |
| ΔINO1 | CEN.PK2-1C; *ΔINO1::loxP* | This study |
| ΔFOX2 | CEN.PK2-1C; *ΔFOX2::loxP* | This study |
| ΔMLS1 | CEN.PK2-1C; *ΔMLS1::loxP* | This study |
| ΔPGC1 | CEN.PK2-1C; *ΔPGC1::loxP* | This study |
| ΔSUT2 | CEN.PK2-1C; *ΔSUT2::loxP* | This study |
| ΔAKR2 | CEN.PK2-1C; *ΔAKR2::loxP* | This study |
| ΔADH5 | CEN.PK2-1C; *ΔADH5::loxP* | This study |
| ΔPSK1 | CEN.PK2-1C; *ΔPSK1::loxP* | This study |
| ΔFUM1 | CEN.PK2-1C; *ΔFUM1::loxP* | This study |
| ΔCIT3 | CEN.PK2-1C; *ΔCIT3::loxP* | This study |
| ΔTKL2 | CEN.PK2-1C; *ΔTKL2::loxP* | This study |
| ΔPGS1 | CEN.PK2-1C; *ΔPGS1::loxP* | This study |
| ΔADR1 | CEN.PK2-1C; *ΔADR1::loxP* | This study |
| ΔHMG2 | CEN.PK2-1C; *ΔHMG2::loxP* | This study |
| ΔSWI1 | CEN.PK2-1C; *ΔSWI1::loxP* | This study |
| ΔSTE14 | CEN.PK2-1C; *ΔSTE14::loxP* | This study |
| ΔPHO2 | CEN.PK2-1C; *ΔPHO2::loxP* | This study |
| ΔDLD3 | CEN.PK2-1C; *ΔDLD3::loxP* | This study |
| ΔTPS13 | CEN.PK2-1C; *ΔTPS13::loxP* | This study |
| ΔPMA2 | CEN.PK2-1C; *ΔPMA2::loxP* | This study |
| ΔARG3 | CEN.PK2-1C; *ΔARG3::loxP* | This study |
| ΔLYS2 | CEN.PK2-1C; *ΔLYS2::loxP* | This study |
| ΔLYS20 | CEN.PK2-1C; *ΔLYS20::loxP* | This study |
| ΔLYS21 | CEN.PK2-1C; *ΔLYS21::loxP* | This study |

**References:**

1. Chen Y, Daviet L, Schalk M, Siewers V, Nielsen J. 2013. Establishing a platform cell factory through engineering of yeast acetyl-CoA metabolism. Metab Eng 15:48-54.http://dx.doi.org/10.1016/j.ymben.2012.11.002. PubMed

2. Chen X, Yang X, Shen Y, Hou J, Bao X. 2018. Screening Phosphorylation Site Mutations in Yeast Acetyl-CoA Carboxylase Using Malonyl-CoA Sensor to Improve Malonyl-CoA-Derived Product. Front Microbiol 9:47.http://dx.doi.org/10.3389/fmicb.2018.00047. PubMed

3. Qiu C, Chen X, Rexida R, Shen Y, Qi Q, Bao X, Hou J. 2020. Engineering transcription factor-based biosensors for repressive regulation through transcriptional deactivation design in *Saccharomyces cerevisiae*. Microb Cell Fact 19:146.http://dx.doi.org/10.1186/s12934-020-01405-1. PubMed

4. Zhang GC, Kong, II, Kim H, Liu JJ, Cate JH, Jin YS. 2014. Construction of a quadruple auxotrophic mutant of an industrial polyploid *saccharomyces cerevisiae* strain by using RNA-guided Cas9 nuclease. Appl Environ Microbiol 80:7694-701.http://dx.doi.org/10.1128/aem.02310-14. PubMed
